# Alpine community recruitment potential is determined by habitat attributes in the alpine ecosystems of the Himalaya‐Hengduan Mountains, SW China

**DOI:** 10.1002/ece3.8373

**Published:** 2021-11-18

**Authors:** Xufang Chen, Lishen Qian, Yazhou Zhang, Honghua Shi, Hang Sun, Jianguo Chen

**Affiliations:** ^1^ Key Laboratory for Plant Diversity and Biogeography of East Asia Kunming Institute of Botany Chinese Academy of Sciences Kunming China; ^2^ University of Chinese Academy of Sciences Beijing China; ^3^ School of Life Sciences Yunnan University Kunming China

**Keywords:** alpine ecosystem, community recruitment, habitat type, Himalaya‐Hengduan Mountain, seed bank, species diversity

## Abstract

The fragility and sensitivity to climate change of alpine ecosystems make it difficult to maintain the stability of their plant communities. Thus, it is important to determine which plant propagules are stored in the soils in order to understand community recruitment potential, especially under different environmental conditions. Based on a soil seed germination and seedling cultivation experiment, we aimed to identify differences in the soil seed attributes between three typical habitat types in the alpine subnival ecosystems of the Himalaya‐Hengduan Mountains and hence to predict the community recruitment potential of each of these different communities. We found that the seed assemblages in the soils differed between habitats. The most abundant taxa were from the genera *Saxifraga*, *Kobresia*, *Arenaria*, *Polygonum*, *Draba*, and *Viola*, while the taxa with lowest abundance were *Apiaceae*, *Campanulaceae*, *Circaea*, *Crassulaceae*, and *Gentiana*. Different habitats exhibited variable soil seed richness, diversity, and density. However, the patterns differed between study sites. Specifically, at Baima (BM) and Shika (SK) snow mountains, soil seed richness, diversity, and density were generally highest in grassland, followed by rock bed and bare ground. In contrast, on Jiaozi (JZ) snow mountain, the rock bed supported the highest soil seed richness and density, followed by grassland and bare ground. These results suggest that the attributes of habitats and communities can both affect the accumulation of soil seeds. Bare ground supports the lowest seed diversity and density but also harbors the most empty niches. We, therefore, predict that, once the thermal conditions become suitable as a result of global warming, this habitat has the potential to see greater changes than grassland and rock bed in terms of community recruitment.

## Graphical abstract

This study identified the differences in the soil seed composition, diversity, and density between three typical habitat types—rock bed, bare ground, and grassland—in the alpine subnival ecosystems on different mountains in the Himalaya‐Hengduan Mountains (HHM). The results not only shed light on the understanding of the current sustainability of alpine communities with different habitat attributes but also contribute a lot to the prediction of potential changes of these communities under future climate warming.

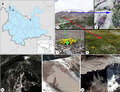

## 1 INTRODUCTION

To persist in natural ecosystems, plant species have to achieve successful recruitment under specific environmental conditions, which are influenced by various factors (Bazzaz, 1991; Burkart et al., 2010; Mayer & Erschbamer, 2011; Song et al., 2013; Tingstad et al., 2015). Recruitment is one of the main challenges for plants in harsh environments, such as alpine and desert ecosystems. In particular, in the alpine subnival ecosystem, the low temperature, poor soil, challenging water conditions, and short growing seasons strictly limit the reproduction, survival, and growth of many alpine plants (Dolezal et al., 2021; Körner, 2003). Generally, the recruitment of plants is determined by the availability, quality, and dispersal ability of their propagules (Scherff et al., 1994; Turnbull et al., 2000; Lindgren et al., 2007; Meineri et al., 2013), the availability of suitable sites (Eriksson & Ehrlén, 1992; Frei et al., 2012; Harper, 1977; Makana & Thomas, 2004; Song et al., 2013), and how many empty niches (i.e., bare ground gaps) are available to be occupied (Ekrtová & Košnar, 2012).

Community recruitment relies, often, on seed germination and seedling establishment (Burkart et al., 2010; Fenner & Thompson, 2005), with the soil seed bank playing an important role (Erfanzadeh et al., 2010; Marone et al., 2004; Thompson, 2000). In fact, in many cases, the soil seed bank is one of the most important components of plant communities, as it acts as a reservoir for potential reproduction to allow the maintenance of communities (Thompson, 2000). Thus, assessing the soil seed bank is fundamental if we are to understand the composition, preservation, conservation, and restoration of plant communities (Erfanzadeh et al., 2010, 2020). Previous studies have suggested that alpine plants employ vegetative propagation as their main reproductive strategy in order to maintain populations (Archibold, 1995; Bliss, 1971); this would mean that propagules other than seeds (clonal reproductive organs) may be present in the soil. Hence, when assessing the recruitment potential in alpine communities, a more comprehensive picture can be obtained by considering all reproductive components, including seeds and nonsexual propagules, in the soil.

**FIGURE 1:**
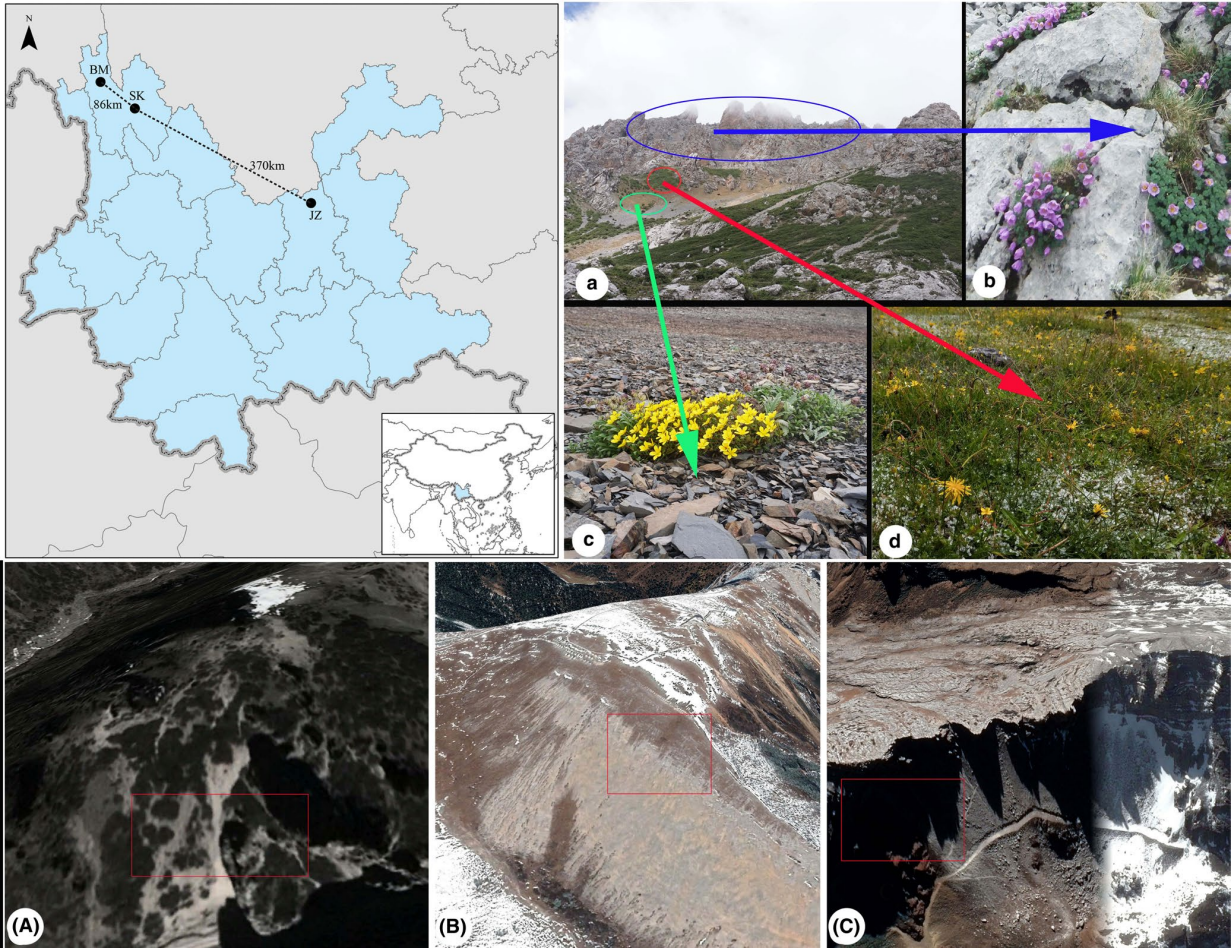
The map of the study sites (top left), landscape of each site (A–C, downloaded from Google earth), and the subnival ecosystem of the Himalaya‐Hengduan Mountains (a–d, photographed by Jianguo Chen). (a) A general view of the subnival ecosystem; (b) a close‐up of the rock bed habitat; (c) a close‐up of the bare ground habitat; (d) a close‐up of the grassland habitat. Note: the close‐ups are not all taken from the same sites shown in a, but the habitat types are more or less the same

In some ecosystems, such as forests, previously established species/individuals occupy most of the local niches thus most seeds in the soil do not germinate; however, when disturbances such as wildfire or grazing occur and new niches (i.e., gaps) are created, seeds from the seed bank can germinate and establish new communities (Coop & Schoettle, 2009; Moen et al., 1993). However, such situations may not exist across most of the alpine subnival communities, as there tend to be many empty niches unoccupied by any species/individuals (Figure 1; also see Dolezal et al., 2021). This could mean that the expansion of plant communities in alpine subnival ecosystems is not limited by the availability of empty niches, but by other factors, such as the availability of a soil seed bank and the suitability of microhabitats. However, few studies have attempted to identify the potential contribution of the soil seed bank to the recruitment of alpine plant communities. Specifically, we are not aware of any study comparing recruitment potential between different habitats in the alpine subnival ecosystem, despite the fact that this could be particularly important for understanding the sustainability of the entire alpine ecosystem.

Although the high Himalaya‐Hengduan Mountains (HHM) experience extreme environmental conditions (Wang, 2006; Zhang et al., 1997), the ecosystems support very high plant diversity (Sun et al., 2017; Xu et al., 2014). To date, 942 plant species, belonging to 168 genera and 48 families, have been recorded in the subnival ecosystem; of these species, 295 (31.32%) are endemic to the Sino‐Himalayan alpine subnival ecosystem and 151 (16.03%) are strictly endemic to the HHM (Xu et al., 2014). Variations in the microclimate and the different weathering processes experienced by the rocks in the alpine subnival ecosystems in this region result in the presence of various types of microhabitat and hence a mosaic of vegetation communities (Figure 1; the comprehensive scientific expedition to the Qinghai‐Xizang plateau & Chinese academy of sciences, 1993; Zhang et al., 1997). Generally, there are three micro‐habitat types in the subnival ecosystems. (a) “Rock bed,” which is commonly found on the top of the mountains. Such rocks are still in the process of weathering and are completely inhospitable to most higher plants, as a result of the absence of soil and available water. However, some pioneer bryophytes and lichens can successfully establish on the surfaces or in the crevices of these rocks. The ability of bryophytes and lichens to modify micro‐environments and create suitable microsites, which can favor plant survival are well‐documented (Beringer et al., 2001; Gornall et al., 2007; Haughian & Burton, 2015; Lett et al., 2020). Consequently, such microsites can offer “safe sites” for seed germination and seedling establishment for some other higher plants after the chance arrival of seeds (Figure 1b). (b) “Bare ground,” which actually is not completely bare, is usually composed of scree and thin soils. Such habitats can offer limited soil and nutrients for plants, but the surface layers are not stable and cannot effectively retain soil moisture and nutrients (Chen, Yang, et al., 2015; Yang et al., 2010); thus, they are also inhospitable to many higher plants (Figure 1c). (c) Grassland or grass shrubland represents a habitat where some stress‐tolerant species, such as cushion plants and shrubs (Chen et al., 2019; Chen, Schöb, et al., 2015; Chen, Yang, et al., 2015; Yang et al., 2010), are able to modify the micro‐environmental conditions, leading to an increase in species numbers and facilitating the formation of numerous vegetation patches of different sizes. Within such patches, the soils develop better and soil moisture and nutrients can be relatively well maintained compared with the surrounding bare ground. As a result, such patches can support more species and thus have higher plant diversity compared with adjacent habitats (Figure 1d). In addition, each of these habitat types is able to support different plant communities, as a result of plants' habitat preferences (Bazzaz, 1991) and/or microsite limitations (Mayer & Erschbamer, 2011; Song et al., 2013).

These three different habitat types are actually distributed within a small area (Figure 1a–d) so that there is a high potential for seed exchange between them unless other limiting factors truly exist. For example, particular moisture regimes are necessary for the germination of each species and for seedling survival, as well as for the successful establishment of mature plant communities in the montane and subalpine zones (Mayer & Erschbamer, 2011). To expand potential distribution, many plants have evolved various special strategies to release and disperse their seeds to increase dispersal distance or to allow them to reach suitable sites (Bansal & Sen, 1981; Donohue, 1997; Fenner & Thompson, 2005; Niu et al., 2016; Willson et al., 2000). Indeed, the seeds of some plants are, thanks to their special functional traits, readily dispersed by wind, water, animals, and human beings (Harper, 1977). In fact, successful seed dispersal coupled with opportunistic seedling growth can allow juveniles to establish in new habitats and overcome a limiting step in the expansion of species' distributions (Scherff et al., 1994). However, the vegetation within each habitat generally forms a natural boundary (Figure 1), indicating that the habitat may truly limit the germination and/or seedling establishment of some species. Nevertheless, few studies have reported on the differences in seed composition between habitats in alpine ecosystems (but see Ekrtová & Košnar, 2012). Examining the differences in the seed composition of distinct microhabitats may help to clarify the relationship between patterns and processes associated with soil seed banks (Marone et al., 2004) and may also reveal the dispersal characteristics of alpine communities, thus allowing us to predict the recruitment potential in alpine communities under future climate warming.

In this study, we aimed to determine the community recruitment potential in different habitat types in the subnival ecosystems of the HHM. We formulated four hypotheses: (a) Different habitats have differing soil seed composition, thus affecting community recruitment potential. Specifically, (b) the microsites created by pioneer bryophytes and lichens in the rock crevices act as “safe sites” for some higher plants; (c) the bare ground habitat has the poorest soil seed bank, as it is directly exposed to strong winds in alpine ecosystems, making it unlikely for seeds to become trapped and retained there; (d) the grassland habitat harbors the highest number of seeds in its soil, as it exhibits the highest plant diversity and most complete vegetation cover, providing the greatest potential to trap seeds.

## 2 MATERIALS AND METHODS

### 2.1 Study site

We selected three study sites in alpine subnival ecosystems in Yunnan province, SW China (Table 1). These were, from north to south, Baima snow mountain (BM), Shika snow mountain (SK), and Jiaozi snow mountain (JZ) (Figure 1). The sites are located in the core area of plant diversity of the Himalaya‐Hengduan Mountains (Zhang et al., 2021), which has been characterized as having a monsoon climate with an annual precipitation between 300 and 1300 mm and annual mean temperature from 0°C to over 20°C (Wang, 2006; Zhang et al., 1997). The soil water and nutrient conditions are quite poor (Zhang et al., 1997). Specifically, the BM site, with an east‐facing slope of 21°, is located in a small mountain valley on Baima snow mountain. A very small stream with water originating from snow melt at higher elevation crosses the valley (Figure 1A). The SK site, with a southwest‐facing slope of 18°, is located close to the ridge of Shika snow mountain (Figure 1B). The JZ site, with a north‐facing slope of 28°, is located close to the ridge of Jiaozi snow mountain (Figure 1C). All sites are on limestone and characterized by freeze–thaw action, cryogenic processes, and large water and temperature variations (Wang et al., 2006). Chen, Yang, et al. (2015), Chen et al. (2019, 2020) found that, at another three sites close to the BM site, soil nutrient concentrations (including organic matter, available nitrogen, phosphorus, and potassium) from underneath shrub and/or cushion patches are significantly higher than those from bare ground. Similar findings have been confirmed from other alpine ecosystems (see review in Padilla & Pugnaire, 2006). As mentioned above, the soils are more developed in the grass and/or grass shrublands, so we assume that soils from grassland or grass shrubland provide better nutrient conditions than bare ground and bed rock at our study sites. In addition, the soil mean temperature 2 cm below ground recorded from late June 2020 to early July 2021 at a site close to the BM site was significantly higher than that at the SK and JZ sites, while there was no difference between the SK and JZ sites [3.63 ± 0.04 (JZ) vs. 3.57 ± 0.03 (SK) vs. 4.23 ± 0.04 (BM), mean ± *SE*; *p* < .001; Chen et al., unpublished data]. We therefore assume that plants at the BM site experience better thermal conditions than at the SK and JZ sites. The vegetation at these three sites is not continuous, and the species composition can change significantly within a short distance (Figure 1). Some species can only survive with the facilitation of other stress‐tolerant species (cushions/shrubs; Chen et al., 2019; Chen, Schöb, et al., 2015; Chen, Yang, et al., 2015; Yang et al., 2010). Some species show a high degree of habitat affinity; for example, *Paraquilegia microphylla* and *Potentilla articulata* prefer to live in the crevices of huge rocks, while some *Saxifraga* species tend to live in scree and *Kobresia* species are more commonly found in grassland (Figure 1).

**TABLE 1:** Characteristics of the study sites and the number of soil samples collected in each habitat type

Study site	Location	Geographic coordinates	Elevation	Slope aspect/gradient	Soil sample no. in rock bed	Soil sample no. in bare ground	Soil sample no. in grassland
BM	NW Yunnan	N28°24′42.277″ E99°1′0.883″	4265 m	South/22°	13	14	18
SK	NW Yunnan	N27°47′25.611″ E99°35′15.032″	4289 m	Southwest/18°	15	15	16
JZ	E Yunnan	N26°9′23.745″ E102°55′38.199″	4070 m	North/28°	10	10	10

### 2.2 Field and laboratory experiments

Fieldwork was conducted late in the growing season (late October) in 2020 when most plants had finished their annual life cycle and seeds had fully matured and been released to the surrounding habitats. At each study site and in each community associated with the different habitat types, that is, rock bed, bare ground, and nearby grassland (or grass shrub) land (Figure 1), we randomly selected a certain number of points (Table 1), according to the availability of habitats, with the distance between any two points more than five meters. At each point, a soil sample of ca. 10 cm × 10 cm × 5 cm was collected after carefully removing the above‐ground plant materials (no visible seeds were removed), but the below‐ground plant tissues were retained as some of them could act as plant propagules (i.e., vegetative reproductive material). Each soil sample was placed in a valve bag in the field and brought back to the laboratory as soon as possible. In the laboratory, soil samples were gently loosened by hand and air‐dried for one month (plants in this area generally experience a short drought period in the early growing season) before being transferred to a greenhouse environment at a temperature of ca. 20–25°C in order to allow the emergence of seedlings (Bourgeois et al., 2017; Chaideftou et al., 2009; Erfanzadeh et al., 2020). Soil samples were spread out in plastic boxes (15 cm × 10 cm × 6 cm), the bottoms of which had several holes of approximately 5 mm diameter drilled in them for drainage. These boxes were placed on larger germination trays. To break any seed dormancy, we initially added 500 ml gibberellin A3 (GA3) solution (Aksenova et al., 2013), at a concentration of 20 mg/L to each of the boxes. We sprayed the trays with sufficient tap water once every three to five days to keep the soils moist during the entire cultivation period. Emerging seedlings were recorded at regular intervals (every five days) until no more new seedlings emerged for at least ten days; we continued observations for about four months. Finally, all seedlings were identified to species level where possible, otherwise to genus or family. Seedlings that could not be identified to species were given a unique ID (e.g., *Aster* sp1.) to make sure that different species were not identified as being the same.

Seedlings could have originated from seeds or vegetative propagules, but we did not distinguish between them, because our aim in this study was to assess community recruitment potential rather than anything else. Irrespective of whether seedlings originate from seeds or vegetative propagules, they both genuinely contribute to community recruitment. However, for ease of presentation, we use “seed” or “seed bank” to refer to all the plant propagules (both seeds and other clonal reproductive organs) found in soils.

### 2.3 Data analysis

Seed density was determined in terms of the number of seedlings per square meter. The Shannon–Wiener index for each sample from each habitat at each study site was estimated using EstimateS v. 9.1.0 software (Colwell, 2013) and selected to allow us to compare the variations in species diversity between different habitat types. In addition, species evenness (*J*) was calculated as *Jn* = *H*′*_n_*/ln(*S_n_*), where ln(*S_n_*) is the maximum diversity that communities could attain if all species were equally abundant, and *n* is the sample size for which values of species diversity (*H*ʹ) and richness (*S*) were used to calculate evenness (Badano & Cavieres, 2006; Magurran, 1988). Linear mixed effects models were constructed to test the difference in species diversity, evenness, and seed density between different habitat types, with study site and habitat type as the fixed factors and sample ID as a random factor.

To compare species richness between different habitats, we constructed species accumulation curves for each habitat type at each study site. For this, we generated a species × samples matrix for each habitat type in which each cell (*i*, *j*) contained a value for either the abundance (number of individuals) or absence (0) of the *i*th species in the *j*th sample. From these matrices, 100 samples were drawn at random, with replacement, for each sample size (ranging from one sample to the maximum number of samples). The estimated values of species richness were then plotted against the respective sample size to construct a sample‐based rarefaction curve for each habitat at each study site. All rarefaction analyses were carried out using EstimateS v. 9.1.0 software (Colwell, 2013).

To examine differences in the composition of the plant species associated with different habitat types, we conducted a distance‐based Redundancy Analysis (dbRDA) with a Bray–Curtis distance metric using the capscale function in the vegan package (Oksanen et al., 2019). The significance of study site, habitat type, and their interaction on the species composition was evaluated with a Monte Carlo permutation test (999 iterations) using the anova.cca function in the vegan package in R 3.5.3 (Oksanen et al., 2019).

## 3 RESULTS

In total, 32, 30, and 23 species germinated from the soils at Baima (BM), Shika (SK), and Jiaozi (JZ) snow mountains, respectively (Table 2). Generally, soils from grassland habitat contained more seeds, followed by soils from rock bed and bare ground habitats for the BM and SK study sites (Table 2). However, for the JZ site, soils from the rock bed contained more seeds than the other two habitats, but as with the other sites the bare ground soils contained the fewest seeds (Table 2). The most abundant taxa were *Saxifraga*, *Kobresia*, *Arenaria*, *Polygonum*, *Draba*, and *Viola*; these taxa were also associated with the highest occurrences (i.e., germinated seedlings in most soil samples), while the taxa with the lowest abundances and fewest occurrences were *Apiaceae*, *Campanulaceae*, *Circaea*, *Crassulaceae*, and *Gentiana*. Some species showed a preference for particular habitat types, but it varied between the three study sites. For example, *Kobresia* sp2 was more abundant in rock bed than in bare ground and grassland at the BM site, while at the SK and JZ sites, it was more abundant in grassland than in rock bed and bare ground (Table 2). Similar patterns were also found for other species, for instance, *Polygonum macrophyllum* and *Saxifraga* sp1 (Table 2). These results indicate that the attributes of each study site (see above) could affect the accumulation of particular species.

**TABLE 2:** Species numbers recorded and the five most frequent species in each habitat type at each study site

Study site	Total species no.	Species no. in rock bed	Five most frequent species	Species no. in bare ground	Five most frequent species	Species no. in grassland	Five most frequent species
BM	32 (316)	17 (99)	*Kobresia sp2* (**28**), *Saxifraga sp3* (**12**), *Arenaria napuligera* (**11**), *Sibbaldia sp1* (**9**), *Polygonum macrophyllum* (**6**)	16 (81)	*Rhodiola sp2* (**19**), *Brassicaceae sp3* (**14**), *Polygonum macrophyllum* (**8**), *Kobresia sp2* (**7**), *Brassicaceae sp4* (**6**)	23 (136)	*Viola sp1* (**16**), *Arenaria napuligera* (**14**), *Polygonum macrophyllum* (**14**), *Brassicaceae sp3* (**10**), *Draba sp2* (**10**)
SK	30 (422)	13 (107)	*Kobresia sp1* (**63**), *Draba sp1* (**13**), *Corydalis sp3* (**8**), *Allium sp1* (**5**), *Polygonum macrophyllum* (**4**)	11 (41)	*Kobresia sp1* (**16**), *Draba sp2* (**7**), *Arenaria barbata* (**6**), *Brassicaceae sp4* (**3**), *Polygonum macrophyllum* (**2**)	18 (274)	*Kobresia sp1* (**199**), *Arenaria napuligera* (**26**), *Viola biflora var*. *Rockiana* (**11**), *Brassicaceae sp4* (**8**), *Apiaceae sp1* (**4**)
JZ	25 (634)	20 (297)	*Saxifraga sp1* (**176**), *Crassulaceae sp1* (**39**), *Kobresia sp1* **(30**), *Kobresia sp2* (**15**), *Corydalis sp1* (**9**)	11 (152)	*Kobresia sp2* (**38**), *Kobresia sp1* (**37**), *Saxifraga sp1* (**37**), *Brassicaceae sp2* (**11**), *Arenaria napuligera* (**9**)	13 (185)	*Kobresia sp2* (**63**), *Saxifraga sp1* (**42**), *Salix brachista* (**24**), *Arenaria napuligera* (**16**), *Polygonum macrophyllum* (**15**)

Note

Numbers in parentheses indicate the individual number of relevant species.

**FIGURE 2:**
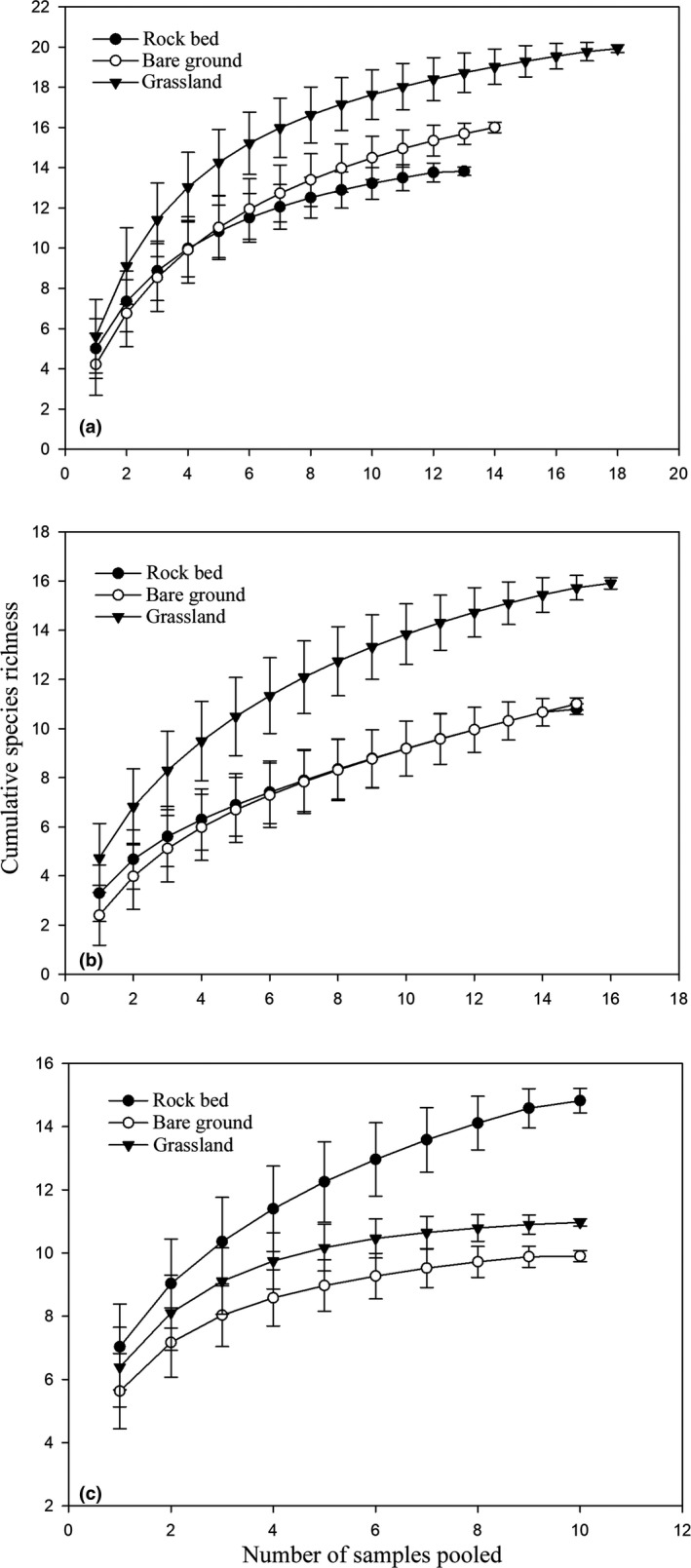
Cumulative species richness estimated from sample‐based rarefaction curves for (a) BM; (b) SK; and (c) JZ study sites

The rarefaction curves (almost) reached asymptotes (Figure 2), indicating that the sampling effort sufficiently captured the composition of seed assemblages in the soils in all habitat types. At the BM and SK sites, species richness was significantly higher in the grassland than in the rock bed and bare ground habitats (Figure 2a,b). Richness in the bare ground habitat tended to increase faster with sampling effort than in the rock bed habitat at the BM site, meaning that species richness in the bare ground habitat was higher than that in rock bed habitat. However, at the SK site, richness in the bare ground and rock bed habitats increased at almost the same rate; that is, the richness in these two habitats was very similar (Figure 2b). Interestingly, site JZ exhibited a completely different pattern of species richness, with the rock bed habitat having the highest species richness, followed by grassland and bare ground (Figure 2c).

**FIGURE 3:**
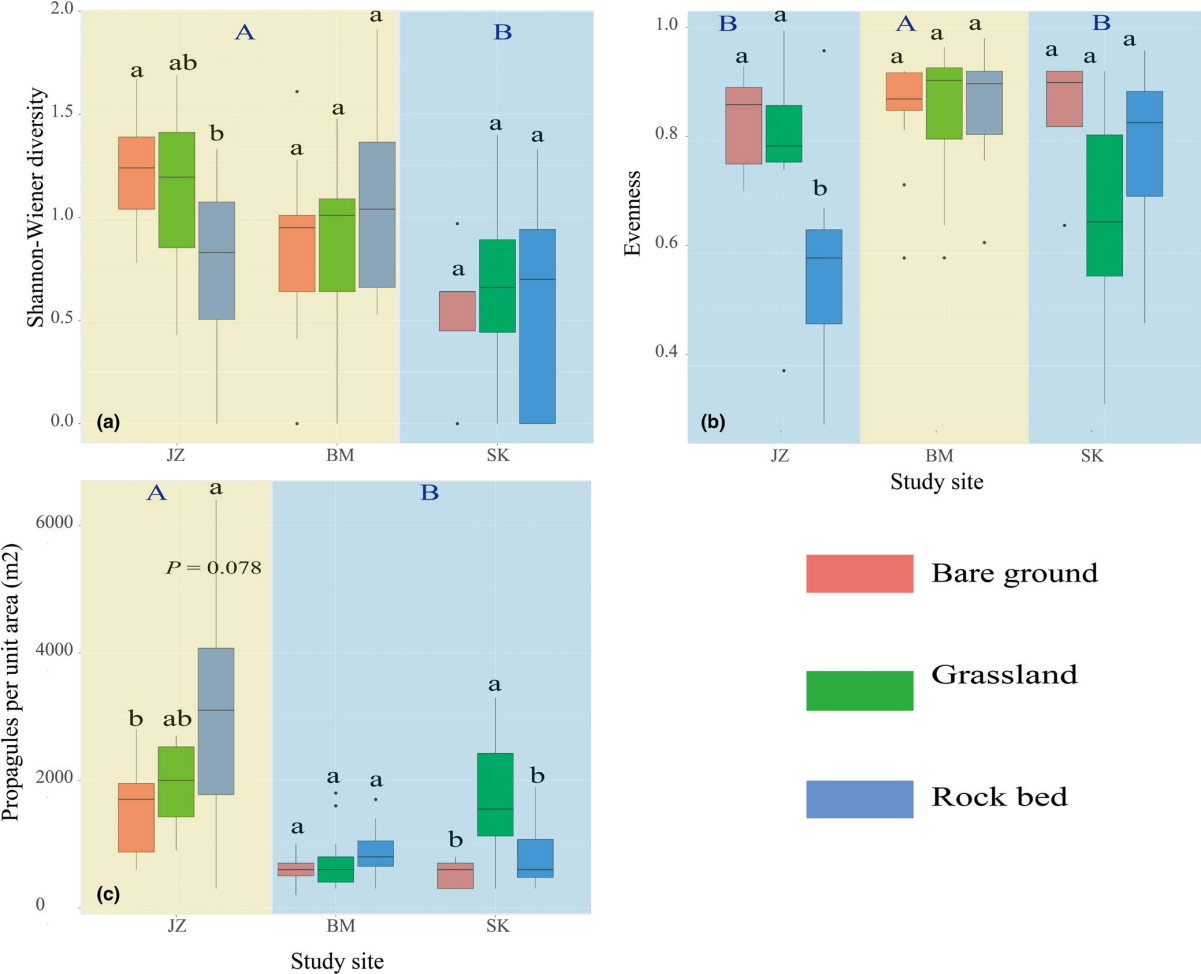
Shannon–Wiener diversity (a), evenness (b), and soil seed density (c) in the different habitats at each study site. Different uppercase characters at the top indicate significant differences between sites and different lowercase characters between habitats

Study site had a significant effect on species diversity, with the BM and JZ sites having higher diversity than SK. In contrast, habitat and the interaction between site and habitat had no effect on species diversity (Table 3; Figure 3a). However, both site and habitat and their interactions had significant effects on species evenness and soil seed density (Table 3). Species evenness was higher at BM than that at SK and JZ (Figure 3b). The evenness was similar in the different habitats at BM and SK, while bare ground and grassland had higher evenness than rock bed at JZ (Figure 3b). Generally, soils from JZ had higher seed density than those from BM and SK, but there was no difference between BM and SK (Figure 3c). In addition, at the SK site, grassland had a higher soil seed density than rock bed and bare ground, while no differences were detected between habitats at BM. However, at JZ, rock bed had the highest soil seed density, followed by grassland and bare ground (Figure 3c).

**TABLE 3:** Results of linear mixed effects models of the effects of site and habitat type on soil seed diversity, evenness and soil seed density

Source	Diversity			Evenness			Seeds/m^2^		
	*df*	*F*	*p*	*df*	*F*	*p*	*df*	*F*	*p*
Site	2	10.32	<.001	2	9.39	<.001	2	24.35	<.001
Habitat type	2	0.27	.77	2	3.24	.04	2	4.87	.01
Site × Habitat type	4	1.84	.13	4	6.06	<.001	4	3.58	.01

**FIGURE 4:**
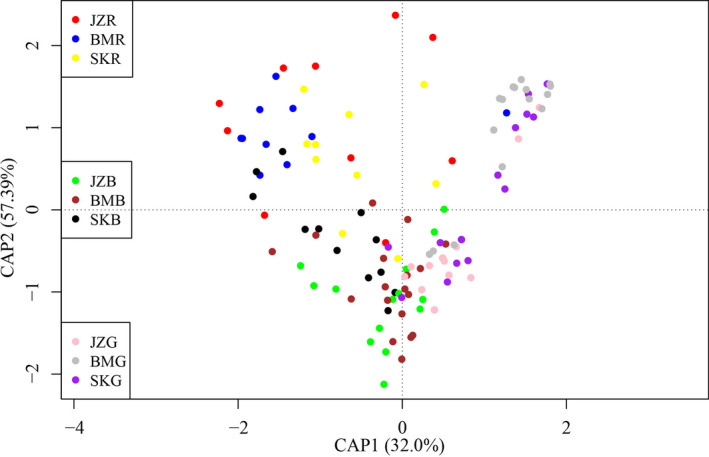
Results of the distance‐based redundancy analysis (dbRDA) with study site and habitat type as the predictor variables. JZ = Jiaozi snow mountain, BM = Baima snow mountain; SK = Shika snow mountain; R = Rock bed; B = Bare ground; G = Grassland

The dbRDA ordination showed that the species composition differed between sites and habitat types, with site (CAP1) and habitat type (CAP2) explaining 89.39% of the total variation (Figure 4), indicating that plants growing under different environmental conditions have different adaptive strategies and thus exhibit habitat affinity to some extent.

## 4 DISCUSSION

Although not the only research in this field, the current work is an important case study examining community recruitment potential in different habitats in alpine ecosystems. Our findings showed that there was different seed composition in the soils of the three habitats, with grassland generally supporting the highest soil seed diversity and density, followed by rock bed and bare ground. However, this pattern was not consistent across the three study sites, possibly due to the micro‐environmental conditions and the regional species pool.

### 4.1 Soil seed composition in different habitats

The total number of species stored in the soils decreased but total number of individuals increased from BM (32 species) to SK (30 species) and then to JZ (23 species) (Table 2). On the one hand, the composition of the soil seed bank is generally positively correlated with the species pool of local communities (Wang et al., 2013). Although we did not investigate the species pool at each study site, our results could suggest that the plant diversity in the surrounding communities exhibits the same trend, that is, decreasing from BM to SK and then to JZ. This is highly likely, considering that the regional species pool can have a strong influence on the local community diversity (species‐pool theory; Eriksson, 1993; Zobel, 1997) and the fact that the total plant diversity in northwestern Yunnan, where BM and SK snow mountains are located, is higher than in other areas (Qian et al., 2020). On the other hand, the higher total abundance at the SK and JZ sites could be attributed to the extensive accumulation of some particular species, for instance *Kobresia* sp1 at SK and *Saxifraga* sp1 at JZ (Table 2). For all three mountains, regardless of habitat type, the most abundant taxa were members of the genera *Saxifraga*, *Kobresia*, *Arenaria*, *Polygonum*, *Draba*, and *Viola*, all with the highest occurrence values. In fact, these taxa were also frequently recorded at our study sites during previous research (Chen et al., 2019; Chen, Schöb, et al., 2015; Chen, Yang, et al., 2015; Yang et al., 2010). Specifically, species like *Saxifraga sinomontana* and *Arenaria barbata* are commonly found growing on alpine screes in the HHM (Chen, Schöb, et al., 2015), while species like *Polygonum macrophyllum* and *Kobresia pygmaea* have broad niche ranges, being found from ca. 2000 m to above 5000 m a.s.l. (http://www.iplant.cn/). On the other hand, the taxa with lowest abundance and fewest occurrences were members of the *Apiaceae*, *Campanulaceae*, *Circaea*, *Crassulaceae*, and *Gentiana*. Some of these taxa, such as *Crassulacea*e and *Gentiana*, are, in fact, frequently present in the alpine ecosystems, but were seldom detected in this study. Besides their low abundance, low reproductive outputs could at least partially account for such results. The low temperature, poor soil water availability, limited nutrient resources, and short growing seasons can generally limit alpine plants' growth and reproduction at all stages (Körner, 2003; Nagy & Grabherr, 2009); as a result, many alpine plants exhibit highly variable reproductive outputs between years (Chambers, 1995), so our suggestion seems reasonable.

Our results indicate that the species composition is different between habitat types (Figure 4), suggesting the occurrence of habitat affinity (Bazzaz, 1991; Scherff et al., 1994) at least for some plant species. For example, in our study area, *Paraquilegia microphylla* and *Potentilla articulata* are strongly associated with rock crevices (Figure 1b), while few individuals of these species can be found in the surrounding bare or grass habitats (personal observation). In addition, such habitat affinity could be affected by the study site, possibly due to the differences in site attributes (see method section). For instance, *Kobresia* sp1 was more abundant in grassland than in bare ground and rock bed at the SK site, while it was more abundant in bare ground than in rock bed at the JZ site, even not belong to the five most frequent species at the BM site (Table 2). Similar patterns could also be found for other taxa. However, we have to note that not all the local soil seeds are necessarily from local mother plants, since longer distance dispersal does occur (Fenner & Thompson, 2005). Many plant seeds are dispersed by wind, animals, and water (Fenner & Thompson, 2005; Harper, 1977) or even by gravity down steep slopes (Erfanzadeh et al., 2020). Different seeds have different dispersal capabilities, resulting in differences in the distances they travel and the habitats that they reach (Fenner & Thompson, 2005). Moreover, different vegetation patches (i.e., micro‐communities) also have different capacities for trapping seeds (Erfanzadeh et al., 2014, 2020; Tessema et al., 2017). Taken together with the effect of habitat attributes (slope for example), it is not surprising that we found different species compositions in different habitat types in this study.

### 4.2 Comparison of soil seed richness, diversity, density, and resultant community recruitment potential between habitats

We found that on the BM and SK mountains, the grassland habitat had the highest soil seed richness (Figure 2), diversity, and density (Figure 3). Such results suggest that grassland may support higher plant diversity, and thus seed sources, than bare ground and rock bed, but these habitats could have different capacities for trapping seeds, with grassland being the most effective and bare ground and rock bed being highly dependent on microsite attributes. Many studies have demonstrated that the attributes of microsites (i.e., vegetation patches) can significantly affect the soil seed characteristics, including composition, diversity, and density (Erfanzadeh et al., 2020; Llorens et al., 2018; Niknam et al., 2018; Raevel et al., 2018). For example, Erfanzadeh et al. (2020) showed that vegetation patches dominated by cushion plants can significantly affect the accumulation of soil seeds; indeed, cushions with different morphological structures also differ in their seed‐trapping capacity. At our study sites, the vegetation in the grassland was generally continuous, at least forming relatively large patches; thus, it should have the greatest capacity to trap seed rain and accumulate the most seeds. Plants can only form very small vegetation patches on rock bed (Figure 1), such patches can trap falling seeds, but the effects are likely to be limited. Compared with grassland and rock bed, very few plants form continuous vegetation patches on bare ground, so seeds deposited in such areas are directly exposed to disturbance by wind and water; this habitat is, therefore, likely to have the most limited capacity to trap and store seeds, as evidenced by the results from this study, especially on JZ mountain, which has the steepest slope (Figure 2).

In terms of soil quality (soil depth, ability to hold soil water and nutrients), the grassland soil is most developed, followed by the bare ground and then rock bed (Figure 1). Higher soil moisture and nutrients can obviously benefit seedling recruitment (Ekrtová & Košnar, 2012; Erschbamer et al., 2001; Mayer & Erschbamer, 2011; Niederfriniger Schlag & Erschbamer, 2000). Previous studies have demonstrated that the modes of seedling recruitment differ significantly between distinct habitats for many plant species (Ekrtová & Košnar, 2012; Garrido et al., 2007; Marone et al., 2004). In addition, seed density in soils is generally higher in natural depressions and under full vegetation cover (trees, shrubs, or grasses) than in open areas between vegetation (Guo et al., 1998; Marone & Horno, 1997). For example, Marone and Horno (1997) found that total seed density was 17 times higher in natural depressions or beneath a full canopy (trees) than in bare soils. Our findings are consistent with such previous findings. Specifically, on the BM and SK mountains, grassland possibly has the highest community recruitment potential since it holds the most soil seeds (Figures 1 and 2). However, whether soil seeds germinate successfully and survive is also dependent on the attributes of the existing vegetation, including vegetation cover, litter thickness, and the abundance of competitors (Lett et al., 2020; Tingstad et al., 2015). In other words, besides abiotic factors, seedling recruitment is also dependent on the availability of empty niches.

It is interesting that, in this study, on JZ mountain, the rock bed has the highest soil seed richness (Figure 2c) and density (Figure 3c), but lower diversity and evenness (Figure 3a,b). From our field observations, it appears that the bryophytes and lichens, which act as efficient seed traps (Sedia & Ehrenfeld, 2003), are better developed on the rock surfaces at JZ than at BM and SK. Furthermore, there are more and larger microcommunities (i.e., vegetation patches) dominated by *Potentilla eriocarpa* on the rock bed on JZ mountain than on BM and SK mountains (Chen et al., unpublished data), which might suggest that the microcommunity patches on JZ mountain have a higher capacity for trapping seeds. As mentioned before, different seeds may have different dispersal abilities, meaning that they travel different distances and reach different habitats (Fenner & Thompson, 2005). Seeds of some species may easily reach and be trapped on the rock bed, while others are unlikely to reach this area; this could explain the high soil seed density but low diversity and evenness. Besides microcommunity patches, other attributes like slope and the distance between continuous vegetation (i.e., seed pool) and the rock bed could also affect the soil seed compositions. However, we are cautious about such explanations, because, unfortunately, we failed to test such effects specifically in this study. Further work is required that is designed to examine the particular reasons behind the differences.

### 4.3 Summary and perspectives

This study demonstrates that the soil seed composition, diversity, and density in the alpine ecosystems of the HHM differ between habitats with varying vegetation composition and cover, indicating that the attributes of habitats can affect the accumulation of seeds in the soil. However, there are some limitations to this study. First, we did not check the seed bank after the germination experiment, so we may have missed some seeds with strong dormancy. To minimize such uncertainty, we added 500 ml GA3 solution (20 mg/L) to the soils in an attempt to break any potential seed dormancy. Second, it is not possible to tell, on the basis of this study, where the seeds originated, making us very cautious when explaining the differences in seed composition between habitats. However, we are most concerned about future community recruitment potential in these different alpine habitats, so the origins of the seeds are, to some degree, of minor importance.

Alpine plant communities whose dynamics are currently largely restricted by low temperature may be highly sensitive to climate warming (Dolezal et al., 2016, 2021). During recent decades, high mountains have warmed much faster than the global average (Pepin et al., 2015). Besides temperature, there are other factors associated with climate warming, such as changes in hydrological and edaphic conditions, which can alter ecosystem processes and the resilience of vegetation (Bellard et al., 2012). Nevertheless, the grassland habitats examined contained the largest seed bank, and there were fewer empty niches (bare patches) than on bare ground and rock bed. Compared with bare ground, the rock bed has the poorest soil conditions, and hundreds of thousands of years of weathering are needed before extensive colonization by plants will be possible. Considering that there are huge niche gaps in the bare ground habitat and long‐distance seed dispersal does occur, it is possible that we might see much greater changes resulting from global warming in this habitat than in grassland and rock bed in terms of community recruitment. However, we did not determine the specific attributes of different habitats (e.g., soil nutrients and moisture) which can influence seedling emergence and survival (e.g., Mayer & Erschbamer, 2011); thus in future, such factors should be specifically determined to make more credible predictions.

## CONFLICT OF INTEREST

The authors declare that they have no conflict of interest.

## AUTHOR CONTRIBUTIONS

**Xufang Chen:** Data curation (supporting); formal analysis (supporting); investigation (lead); writing‐review & editing (supporting). **Lishen Qian:** Data curation (supporting); investigation (supporting); visualization (supporting); writing‐review & editing (supporting). **Yazhou Zhang:** Formal analysis (supporting); investigation (supporting); visualization (supporting); writing‐review & editing (supporting). **Honghua Shi:** Investigation (supporting); visualization (supporting); writing‐review & editing (supporting). **Hang Sun:** Conceptualization (equal); funding acquisition (lead); resources (equal); writing‐original draft (supporting); writing‐review & editing (supporting). **Jianguo Chen:** Conceptualization (lead); formal analysis (lead); funding acquisition (supporting); investigation (lead); visualization (lead); writing‐original draft (lead); writing‐review & editing (lead).

## Data Availability

Data pertaining to the species are available in the Dryad Digital Repository: https://doi.org/10.5061/dryad.5mkkwh774.
